# In vivo characterization of [^18^F]AVT-011 as a radiotracer for PET imaging of multidrug resistance

**DOI:** 10.1007/s00259-019-04589-w

**Published:** 2019-11-15

**Authors:** Pavitra Kannan, András Füredi, Sabina Dizdarevic, Thomas Wanek, Severin Mairinger, Jeffrey Collins, Theresa Falls, R. Michael van Dam, Divya Maheshwari, Jason T. Lee, Gergely Szakács, Oliver Langer

**Affiliations:** 1 CRUK and MRC Oxford Institute for Radiation Oncology, University of Oxford, Oxford, UK; 2 Department of Microbiology, Tumor and Cell Biology, Karolinska Institutet, Stockholm, Sweden; 3 Institute of Enzymology, Research Centre for Natural Sciences, Hungarian Academy of Sciences, Budapest, Hungary; 4 Institute of Cancer Research, Medical University of Vienna, Vienna, Austria; 5 Brighton and Sussex University Hospitals, NHS Trust and Brighton and Sussex Medical School, Brighton, UK; 6 Preclinical Molecular Imaging, AIT Austrian Institute of Technology GmbH, Seibersdorf, Austria; 7 Crump Institute for Molecular Imaging and Department of Molecular & Medical Pharmacology, David Geffen School of Medicine at UCLA, Los Angeles, CA USA; 8 Jonsson Comprehensive Cancer Center, David Geffen School of Medicine at UCLA, Los Angeles, CA USA; 9 Avaant Imaging, Lexington, MA USA; 10 Stanford Center for Innovations in In vivo Imaging, Stanford University School of Medicine, Stanford, CA USA; 11 Department of Clinical Pharmacology, Medical University of Vienna, Vienna, Austria; 12 Department of Biomedical Imaging und Image-guided Therapy, Division of Nuclear Medicine, Medical University of Vienna, Vienna, Austria

**Keywords:** Cancer, Multidrug resistance, PET imaging, ABCB1, ABCG2

## Abstract

**Purpose:**

Multidrug resistance (MDR) impedes cancer treatment. Two efflux transporters from the ATP-binding cassette (ABC) family, ABCB1 and ABCG2, may contribute to MDR by restricting the entry of therapeutic drugs into tumor cells. Although a higher expression of these transporters has been correlated with an unfavorable response to chemotherapy, transporter expression does not necessarily correlate with function. In this study, we characterized the pharmacological properties of [^18^F]AVT-011, a new PET radiotracer for imaging transporter-mediated MDR in tumors.

**Methods:**

AVT-011 was radiolabeled with ^18^F and evaluated with PET imaging in preclinical models. Transport of [^18^F]AVT-011 by ABCB1 and/or ABCG2 was assessed by measuring its uptake in the brains of wild-type, *Abcb1a/b*^−/−^, and *Abcg2*^−/−^ mice at baseline and after administration of the ABCB1 inhibitor tariquidar (*n* = 5/group). Metabolism and biodistribution of [^18^F]AVT-011 were also measured. To measure ABCB1 function in tumors, we performed PET experiments using both [^18^F]AVT-011 and [^18^F]FDG in mice bearing orthotopic breast tumors (*n* = 7–10/group) expressing clinically relevant levels of ABCB1.

**Results:**

At baseline, brain uptake was highest in *Abcb1a/b*^−/−^ mice. After tariquidar administration, brain uptake increased 3-fold and 8-fold in wild-type and *Abcg2*^−/−^ mice, respectively, but did not increase further in *Abcb1a/b*^−/−^ mice. At 30 min after injection, the radiotracer was > 90% in its parent form and had highest uptake in organs of the hepatobiliary system. Compared with that in drug-sensitive tumors, uptake of [^18^F]AVT-011 was 32% lower in doxorubicin-resistant tumors with highest ABCB1 expression and increased by 40% with tariquidar administration. Tumor uptake of [^18^F]FDG did not significantly differ among groups.

**Conclusion:**

[^18^F]AVT-011 is a dual ABCB1/ABCG2 substrate radiotracer that can quantify transporter function at the blood-brain barrier and in ABCB1-expressing tumors, making it potentially suitable for clinical imaging of ABCB1-mediated MDR in tumors.

**Electronic supplementary material:**

The online version of this article (10.1007/s00259-019-04589-w) contains supplementary material, which is available to authorized users.

## Introduction

Two efflux transporters from the ATP-binding cassette (ABC) family, ABCB1 and ABCG2, are thought to contribute to multidrug resistance (MDR) in cancer [1]. While these transporters help protect the body from exposure to toxins in physiological conditions, they recognize a large number of anticancer agents as substrates and prevent their entry into tumor cells [2]. In clinical studies, the expression of *ABCB1/ABCG2* mRNA has been shown to inversely correlate with response to chemotherapy in hematological malignancies [3–5] and solid tumors [6]. Tumors refractory to chemotherapy also have an increased mRNA expression of *ABCB1* and/or *ABCG2* [3, 7, 8].

However, upregulation of mRNA expression in treatment-refractory cancer does not necessarily imply that the transporters confer clinical MDR. Only in a few cancers is the expression of ABCB1 and/or ABCG2 proteins higher than that found in normal tissues [9, 10]. Furthermore, clinical trials using ABCB1 inhibitors to increase tumor uptake of chemotherapeutics had little success, partly because patients were not always screened for MDR status and partly because ABCB1 is not known to mediate MDR in some of the tested cancers [1, 11]. Yet, MDR remains a major impediment in cancer treatment [1], and current clinical practice does not screen for MDR status or the development of MDR during therapy. A method that identifies patients who might have drug-resistant tumors due to transporters could potentially improve clinical management of MDR by allowing oncologists to personalize drug selection and predict clinical response [1].

Measurements of transporter function, as opposed to mRNA detection, may detect transporter-mediated MDR and thereby predict therapeutic response [12]. Positron emission tomography (PET) or single-photon emission computed tomography (SPECT) imaging can non-invasively assess the function of ABCB1 in vivo [11], which may provide insight into how this transporter contributes to MDR in patients [1]. Typically, the washout rate and tumor uptake of radiolabeled substrates have been used as measures of transporter activity. For example, in both mouse and human breast tumors, the washout rate of the SPECT radiotracer [^99m^Tc]sestamibi was 2- to 5-fold higher in tumors with high levels of ABCB1 than in those with basal levels [13–16]. Tumor uptake of substrate radiotracers, including [^99m^Tc]sestamibi, [^18^F]fluoropaclitaxel, [^11^C]verapamil, and (*R*)-[^11^C]verapamil, has also been used as a marker of ABCB1 function in drug-resistant cancers [17–20]. However, these substrate radiotracers suffer from drawbacks, including lower quantitative accuracy of SPECT as compared with PET ([^99m^Tc]sestamibi), low signal ratio (1.1–1.3) between tumors with basal and high ABCB1 levels ([^18^F]fluoropaclitaxel, [^11^C]verapamil, and (*R*)-[^11^C]verapamil), and extensive radiometabolism ([^18^F]fluoropaclitaxel, [^11^C]verapamil, and (*R*)-[^11^C]verapamil) [17, 20]. The ABCB1 inhibitor tariquidar was also radiolabeled for PET imaging of ABCB1 in tumors [20, 21], but there is conflicting evidence whether it is transported by ABCB1 [22, 23].

A PET substrate radiotracer with an ^18^F-label that addresses some of these drawbacks would improve quantification of transporter-mediated MDR. Here, we evaluated the pharmacological characteristics of a new ^18^F-labeled PET radiotracer, [^18^F]AVT-011 ([2-(4-{2-[6-(2-[^18^F]fluoroethoxy)-7-methoxy-3,4-dihydro-1*H*-isoquinolin-2-yl]ethyl}phenylcarbamoyl)-4,5-dimethoxyphenyl]amide), for imaging MDR mediated by ABC transporters in vivo. We developed a two-step radiosynthesis procedure, and evaluated if the radiotracer is transported by ABCB1 and ABCG2. We then tested its ability to detect ABCB1-mediated MDR in vivo by measuring its uptake in an orthotopic model of chemotherapy-resistant breast cancer expressing clinically relevant levels of ABCB1.

## Materials and methods

### Chemicals and radiochemistry

Details regarding chemicals, inhibitor formulation, and synthesis of [^18^F]AVT-011 are described in Supplemental Methods. Synthesis was performed at two different sites: University of California Los Angeles (UCLA) and Austrian Institute of Technology (AIT).

### Animal models

For substrate selectivity studies, female mice from three models were used: wild-type (*n* = 5 mice; 25.2 ± 2.1 g; model FVB), *Abcb1a/b*^*−/−*^ (*n* = 5 mice; 22.3 ± 2.1 g), and *Abcg2*^*−/−*^ (*n* = 5 mice; 21.3 ± 1.5 g; Taconic Biosciences, USA). For tumor uptake studies, female wild-type mice were used (*n* = 27 mice; 24.7 ± 2.3 g, model FVB; Envigo). Tumors expressing different levels of ABCB1 were generated from *Brca1*^*−*/*−*^;*p53*^*−*/*−*^ FVB mouse mammary tumors as described before [24, 25]. Briefly, orthotopic transplants were generated by re-transplanting tumor pieces derived from the *Brca1*^*−*/*−*^;*p53*^*−*/*−*^ model into the mammary fat pads of wild-type mice to generate three groups of animals bearing tumors with basal, intermediate, or high levels of ABCB1. The tumors with intermediate and high ABCB1 levels were treated with doxorubicin (5 mg/kg i.v.) or with pegylated liposomal doxorubicin (PLD, 8 mg/kg i.v.), respectively [26]. Thawed pieces were used for re-transplantation. Tumor volume was measured by calipers using the $V=\frac{\pi }{6}\times l\times {w}^2$ formula, where *l* is the tumor length and *w* is the tumor width. PET imaging was performed on tumors with volumes of 500–1000 mm^3^.

Animal experiments were conducted in accordance with approved protocols and guidelines from the Chancellor’s Animal Research Committee at UCLA or in accordance with the European Communities Council Directive (2010/63/EU), using approved protocols from the Amt der Niederösterreichischen Landesregierung.

### PET imaging of transport activity at the blood-brain barrier

To assess whether [^18^F]AVT-011 was a substrate of mouse ABCB1 and ABCG2, we quantified its brain uptake in wild-type, *Abcb1a/b*^*−/−*^, and *Abcg2*^*−/−*^ mice with PET, before and after treatment with tariquidar, an ABCB1 inhibitor. Mice were anesthetized using 2% isoflurane in oxygen and cannulated on the lateral tail vein. For inhibition studies, mice were injected i.v. with 15 mg/kg tariquidar 30 min prior to injection of [^18^F]AVT-011 (4.9 ± 2.3 MBq). PET images were acquired on the G8 PET/CT (Sofie Biosciences), Inveon PET (Siemens Medical Solutions), or microPET Focus 220 (Siemens Medical Solutions) calibrated scanner. Whole body PET/CT images were acquired simultaneously in listmode for 120 min with a sequence of 6 × 10 s, 4 × 60 s, 5 × 300 s, and 9 × 600 s.

### PET imaging of transporter activity in MDR tumors

The ability of [^18^F]AVT-011 to detect ABCB1-mediated MDR in tumors was assessed by PET imaging of its uptake in breast tumors implanted orthotopically in mice. Whole body PET imaging with [^18^F]AVT-011 was performed using a microPET Focus 220 camera. Mice were anesthetized with 2–3.5% isoflurane and injected via the tail vein with [^18^F]AVT-011 (5.3 ± 2.1 MBq; 0.1 mL). One cohort of mice underwent 90-min dynamic PET scans with [^18^F]AVT-011. During imaging, at 45 min after [^18^F]AVT-011 injection, tariquidar (15 mg/kg) was administered as a short i.v. bolus over 1 min. PET data acquisition was then continued until 90 min after radiotracer injection. Another cohort of animals bearing tumors from the basal and high ABCB1–expressing groups underwent 90-min dynamic PET scans without tariquidar injection. To determine whether tariquidar administration potentially affected the kinetics of [^18^F]AVT-011 in tumor tissue by another mechanism than ABCB1 inhibition, we compared uptake in muscle tissue (a control region without ABCB1 expression) of animals that received a mid-scan tariquidar injection versus those that did not (*n* = 3 mice/group).

For anatomical localization of tumors, [^18^F]FDG PET scans were acquired the day before [^18^F]AVT-011 PET imaging. Mice were fasted for a minimum of 6 h prior to [^18^F]FDG injection. Mice had access to drinking water ad libitum. [^18^F]FDG (5.7 ± 0.6 MBq, 0.1 mL) was injected intraperitoneally and a 15-min static PET emission scan was initiated 60 min after [^18^F]FDG administration. Attenuation correction was performed for tumor imaging. A 10-min transmission scan using a rotating ^57^Co-source was acquired prior initiation of the emission scans. Emission scans were performed with an energy window of 250–750 keV and a timing window of 6 ns.

### Metabolite and biodistribution analysis

Radiometabolism and biodistribution of [^18^F]AVT-011 were measured ex vivo as described in Supplemental Methods.

### Data analysis

PET data were decay-corrected and normalized to units of percent injected dose per gram (%ID/g), and tissue time-activity curves were generated using AMIDE [27] as described in Supplemental Methods.

### mRNA and protein quantification in tumors

Tumor expression of *Abc1a/b* was assessed using qPCR, while ABCB1 levels were measured using Western blotting as described in Supplemental Methods. Gene names are indicated by italics (human: *ABCB1*, *ABCG2*; mouse: *Abcb1a/b*, *Abcg2*), while protein names are indicated in non-italic, capital letters (human: ABCB1; mouse: ABCB1A, ABCB1B; human/mouse: ABCG2).

### Statistical analysis

Statistical differences between two groups were analyzed by two-tailed unpaired *t* tests while differences between multiple groups were analyzed by 1-way, 2-way ANOVA, or Kruskal-Wallis, and corrected for multiple comparisons with Dunnett’s or Dunn’s test, respectively, using the Prism 7 software (GraphPad Software, USA). All values are given as mean ± standard deviation (SD), except for relative mRNA levels, given as mean ± standard error of the mean (SEM).

## Results

### AVT-011 can be labeled with ^18^F

**Fig. 1:**
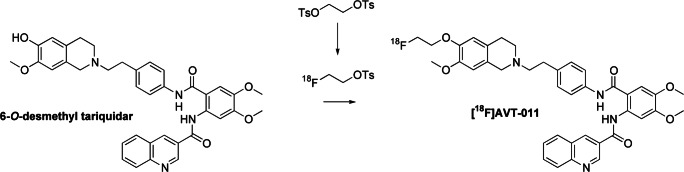
Reaction scheme for the synthesis of [^18^F]AVT-011

^18^F-AVT-011 was produced at two different sites (UCLA and AIT) using a two-step reaction involving (i) the synthesis of [^18^F]fluoroethyl-tosylate by nucleophilic reaction of cyclotron-produced [^18^F]fluoride with ethylene di(p-toluenesulfonate) followed by (ii) reaction of [^18^F]fluoroethyl-tosylate with 6-*O*-desmethyl tariquidar (Fig. 1). Crude product was purified by semipreparative HPLC. The decay-corrected radiochemical yields of [^18^F]AVT-011 based on starting [^18^F]fluoride were 3.7 ± 1.0% (*n* = 4 syntheses) at UCLA and 1.1 ± 0.6% (*n* = 8 syntheses) at AIT. Radiochemical yields were not optimized but were sufficiently high for preclinical imaging. However, further optimization would be required for clinical applications, such as the performance of a single step [^18^F]fluorination reaction using an appropriate tosylate precursor molecule. The total synthesis time was approximately 95 min. Molar activities and radiochemical purities of [^18^F]AVT-011 at the end of synthesis were 143 ± 31 GBq/μmol and 95 ± 2% (*n* = 4) at UCLA, and 99 ± 39 GBq/μmol and 96 ± 3% at AIT. The identity of [^18^F]AVT-011 was verified by HPLC co-injection with unlabeled AVT-011 (Supplemental Fig. 1).

### [^18^F]AVT-011 is a substrate of mouse ABCB1A/B and ABCG2

**Fig. 2:**
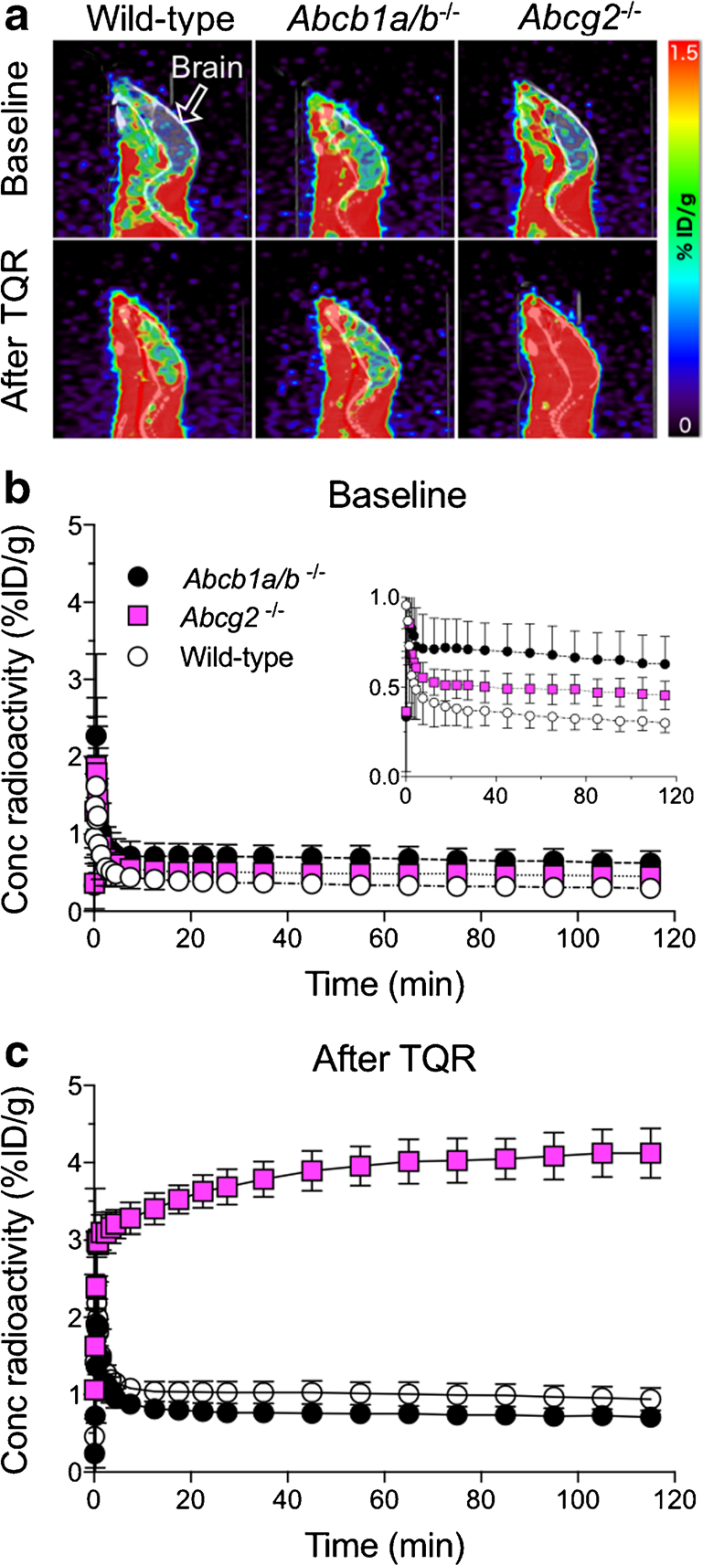
[^18^F]AVT-011 is a substrate of mouse ABCB1 and ABCG2, as assessed by PET imaging. **a** Representative summation images of radiotracer uptake in the brain (% injected dose, (ID)/g) after intravenous injection of [^18^F]AVT-011 in wild-type or transgenic mice lacking *Abcb1a/b* or *Abcg2*. **b** Time-activity curves in all strains of mice at baseline conditions and **c** after pharmacological inhibition of ABCB1 with 15 mg/kg tariquidar (TQR). Symbols represent mean ± SD from *n* = 5 mice/strain, with the exception of *Abcg2*^*−/−*^ after inhibition (*n* = 2). Inset shows time-activity curve ranging from 0 to 1 %ID/g

To determine whether [^18^F]AVT-011 is a substrate of mouse ABCB1 and/or ABCG2, we assessed its uptake in the brains of wild-type and transgenic mice lacking either transporter (Fig. 2a). The brain was selected as the region to assess selectivity because both transporters are co-expressed at the blood-brain barrier and prevent substrate entry into the brain. The uptake of [^18^F]AVT-011 in brain peaked within the first 5 min before reaching a plateau for the remaining scan time (Fig. 2b). Brain uptake (area under the curve, AUC_5–120 min_) of [^18^F]AVT-011 was 2-fold higher in *Abcb1a/b*^*−*/*−*^ mice (*P*_adj_ < 0.01) than in wild-type mice but was not increased in *Abcg2*^*−*/*−*^ mice (*P*_adj_ = 0.14) (Table 1). After pretreatment with tariquidar (15 mg/kg), brain uptake of [^18^F]AVT-011 increased 3-fold in wild-type mice (*P*_adj_ < 0.001) and 8-fold in *Abcg2*^*−*/*−*^ mice (*P*_adj_ < 0.001), relative to baseline values. However, uptake did not significantly increase in *Abcb1a/b*^*−*/*−*^ mice after inhibition (*P*_adj_ = 0.78; Fig. 2c and Table 1).

**Table 1:** Brain uptake of radioactivity measured with PET in three strains of mice after injection of [^18^F]AVT-011 at baseline and after pharmacological inhibition of ABCB1A/B

	Brain uptake (%ID/g*min)		
Condition	Wild-type	*Abcb1a/b*^−/−^	*Abcg2*^−/−^
Baseline	0.37 ± 0.08	0.73 ± 0.17**	0.53 ± 0.09
After TQR	1.08 ± 0.15	0.81 ± 0.11*	4.19 ± 0.28***

Brain uptake (%ID/g*min) was quantified as the area under the time-activity curve from 5 to 120 min after injection of [^18^F]AVT-011. Tariquidar (TQR) was administered 30 min prior to injection of the radiotracer. Data represent mean ± SD from *n* = 5 mice/group; *n* = 2 for *Abcg2*^*−/−*^ after tariquidar. *P_adj_ < 0.05, **P_adj_ < 0.01, and ***P_adj_ < 0.001, against wild-type, using two-way ANOVA with Dunnett’s correction

### [^18^F]AVT-011 is minimally metabolized and predominantly undergoes hepatobiliary excretion

We then assessed the radiometabolism and biodistribution of [^18^F]AVT-011. At 30 min after injection of [^18^F]AVT-011, the percentage of intact parent radiotracer measured in mouse liver was 93.3 ± 2.8% in wild-type mice (*n* = 3), 93.6 ± 1.7% in *Abcb1a/b*^*−*/*−*^ mice (*n* = 4), and 94.0 ± 1.4% in *Abcg2*^*−*/*−*^ mice (*n* = 2), with 84.0 ± 5.0% of radioactivity recovered from liver tissue. Apart from the blood-brain barrier, ABCB1 and ABCG2 are also expressed in the kidneys, liver, small intestine, and lungs [11]. Analysis of organ uptake from PET scans revealed that the small intestine, spleen, liver, and kidney had the highest values in all three strains of mice (Table 2). Uptake in organs was not significantly different among the strains, except in the spleen and small intestine. In the spleen, radioactivity concentrations were 27% higher in *Abcb1a/b*^*−*/*−*^ mice (*P*_adj_ = 0.01) and 51% lower in *Abcg2*^*−*/*−*^ mice (*P*_adj_ < 0.001) than in wild-type mice. In the small intestine, concentrations were 60% and 75% lower in *Abcb1a/b*^*−*/*−*^ and *Abcg2*^*−*/*−*^ mice (*P*_adj_ < 0.001) than in wild-type mice. Ex vivo biodistribution measurements also showed highest uptake in the hepatobiliary system and the kidney of all three strains (Supplemental Table 1). Uptake was also 67% higher in the lungs of *Abcb1a/b*^*−*/*−*^ and *Abcg2*^*−*/*−*^ mice (*P*_adj_ < 0.001) than in those of wild-type mice (Supplemental Table 1). Although brain uptake was not statistically different among groups in ex vivo biodistribution measurements (likely because ex vivo data lacked statistical power due to smaller group sizes), effect sizes among groups were similar to those obtained in vivo.

**Table 2:** Uptake of radioactivity measured with PET in various organs from three strains of mice after injection of [^18^F]AVT-011

	Organ uptake (%ID/g*min)		
Organ	Wild-type	*Abcb1a/b*^−/−^	*Abcg2*^−/−^
Bladder	11.2 ± 1.6	9.3 ± 3.1	9.6 ± 2.0
Heart	1.7 ± 0.2	2.0 ± 0.1	1.9 ± 0.1
Kidney	12.5 ± 0.9	12.2 ± 1.0	11.7 ± 0.3
Liver	14.7 ± 0.8	13.1 ± 0.6	18.0 ± 0.7
Muscle	1.3 ± 0.1	1.8 ± 0.1	2.0 ± 0.2
Small intestine	36.3 ± 10.6	14.5 ± 0.7***	9.0 ± 1.4***
Spleen	23.5 ± 7.0	29.9 ± 8.7*	11.5 ± 1.8***

Organ uptake (%ID/g*min) was quantified as the area under the time-activity curve from 5 to 120 min after injection of [^18^F]AVT-011. Data represent mean ± SD from *n* = 5 mice/group. *P_adj_ < 0.05 and ***P_adj_ < 0.001, against wild-type, using two-way ANOVA with Dunnett’s correction

### [^18^F]AVT-011 can image ABCB1 function in chemotherapy-resistant tumors

**Fig. 3:**
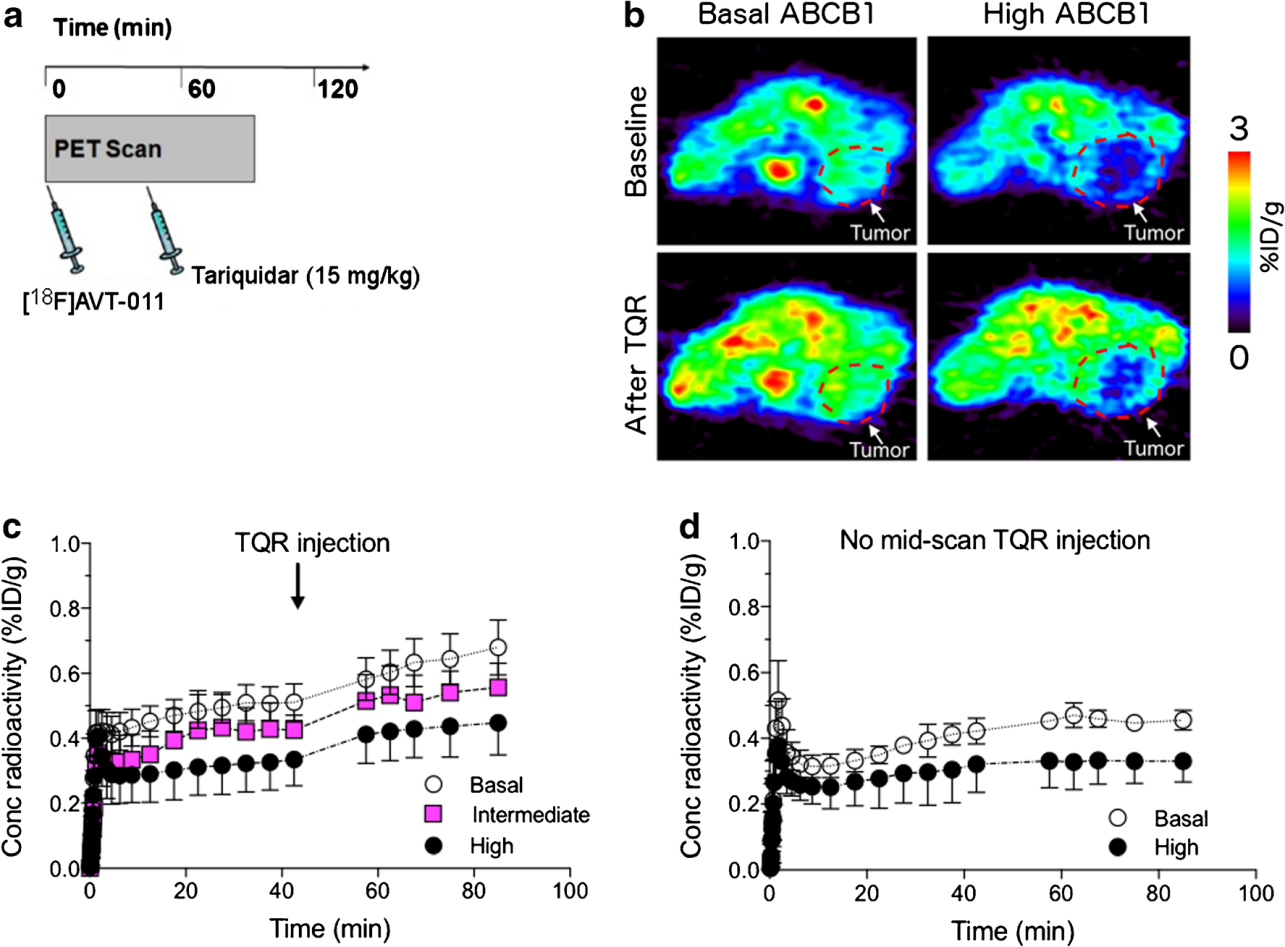
[^18^F]AVT-011 can detect ABCB1 function in an orthotopic mouse model of breast cancer, as assessed by PET imaging. **a** Schematic of dynamic PET study performed in tumor-bearing mice. **b** Summation PET images of [^18^F]AVT-011 from 0 to 45 min (baseline) and from 45 to 90 min (tariquidar, TQR) after radiotracer injection in basal and high ABCB1–expressing tumor groups. Color bar represents radioactivity concentration (%ID/g), set from 0.2 to 2. Anatomical structures are highlighted with white broken lines and labeled with arrows. **c** Time-activity curves in tumors expressing basal, intermediate, and high levels of ABCB1. Tariquidar (TQR, 15 mg/kg) was injected intravenously at 45 min. Symbols represent mean ± SD from *n* = 6 mice (basal tumors), *n* = 7 mice (intermediate-ABCB1 tumors), and *n* = 5 (high-ABCB1 tumors). **d** Time-activity curves in tumors expressing basal and high levels of ABCB1 not treated with tariquidar. Symbols represent mean ± SD from *n* = 3 mice (basal tumors) and *n* = 5 mice (high-ABCB1 tumors)

**Fig. 4:**
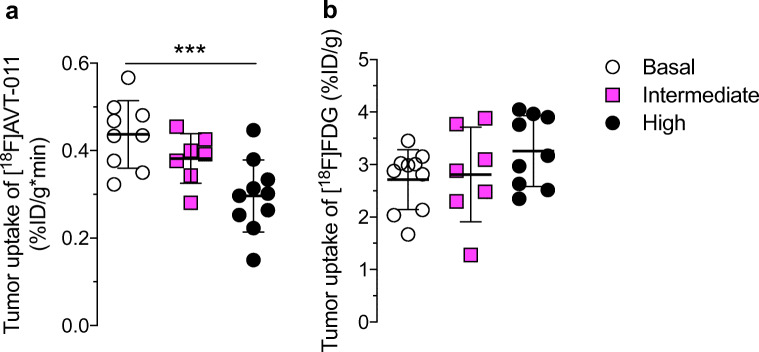
[^18^F]AVT-011 can discriminate tumors expressing basal and high levels of ABCB1, while [^18^F]FDG cannot. **a** Tumor uptake of [^18^F]AVT-011, measured as the area under the curve of %ID/g from 5 to 42.5 min, in tumors expressing basal (*n* = 9), intermediate (*n* = 7), or high (*n* = 10) levels of ABCB1. **b** Tumor uptake of [^18^F]FDG, measured as %ID/g at 60 min after injection, in the same tumors imaged 1 day prior to [^18^F]AVT-011. ***P < 0.001 by one-way ANOVA followed by Dunnett’s multiple comparisons test

To measure ABCB1 function in chemotherapy-resistant tumors, we imaged three groups of mice with [^18^F]AVT-011 before and after tariquidar treatment (Fig. 3a, b) and with [^18^F]FDG for anatomical localization. Wild-type mice were implanted orthotopically with breast tumors comprising three levels of *Abcb1a/b* expression and ABCB1 protein levels (Supplemental Fig. 2) found in clinical tumors [1]: basal, intermediate (3-fold increase in *Abcb1a/b*), or high (~ 521-fold increase in *Abcb1a/b*). These tumor grafts were derived from *Brca1*^*−*/*−*^;*p53*^*−*/*−*^ mouse breast tumors that were previously responsive to chemotherapy (basal) or developed resistance after several cycles of treatment with doxorubicin (intermediate) or with pegylated liposomal doxorubicin [26]. After injection of [^18^F]AVT-011, radioactivity concentrations (%ID/g) in tumors peaked within the first 5 min and reached a plateau by 45 min (Fig. 3c, d). Tumor uptake of [^18^F]AVT-011, measured as AUC of %ID/g*min from 5–42.5 min, was 15% lower in the intermediate group (*P*_adj_ = 0.10) and 32% lower in the high ABCB1–expressing group (*P* < 0.001) than in the basal group (Fig. 4a). In contrast, tumor uptake of [^18^F]FDG, measured as %ID/g at 60 min after injection, was not significantly different among the three groups (intermediate vs basal, *P*_adj_ = 0.92; high vs basal, *P*_adj_ = 0.16; Fig. 4b).

Radioactivity concentrations in the tumor increased in all groups following a bolus injection of tariquidar (15 mg/kg) administered 45 min after injection of [^18^F]AVT-011 (Fig. 3c). Relative to tumor uptake at 45 min, tumor uptake (%ID/g) at 90 min increased by 27.5 ± 9.7% in the basal group, by 31.2 ± 13.3% in the intermediate group, and by 40.9 ± 3.7% in the high ABCB1–expressing group. The percentage increase in the high ABCB1–expressing group was significantly higher than in the basal group (*P*_adj_ = 0.02). By contrast, the increase in tumor uptake between 45 and 90 min in animals not treated with tariquidar (Fig. 3d) was 10.4 ± 10.6% in the basal group (*n* = 4 mice) and 5.4 ± 12.6% in the high ABCB1–expressing group (*n* = 5 mice). The percentage increase in muscle uptake (%ID/g) at 90 min after radiotracer injection relative to muscle uptake at 45 min (before tariquidar administration) was not significantly different between animals injected with tariquidar mid-scan (24.1 ± 5.0 %) and those not injected with tariquidar mid-scan (29.1 ± 2.9%, Supplemental Fig. 3). At the end of the scan (i.e., at 90 min), intact [^18^F]AVT-011 in mouse plasma was 83.8 ± 1.9% in the basal group, 54.1 ± 14.8% in the intermediate group (*P*_adj_ = 0.02), and 85.7 ± 2.6% in the high ABCB1–expressing group (*P*_adj_ = 0.83).

## Discussion

### Substrate selectivity of [^18^F]AVT-011

We characterized the pharmacological properties of [^18^F]AVT-011and tested its utility as a radiotracer for imaging ABC transporter function in MDR tumors. [^18^F]AVT-011 is a substrate of mouse ABCB1A/B, evidenced by the 2-fold enhanced uptake in the brains of *Abcb1a/b*^−/−^ mice relative to wild-type mice at baseline, and by the increased uptake in the brains of wild-type and *Abcg2*^−/−^ mice after administration of tariquidar, which predominantly inhibited ABCB1 and not ABCG2 at the employed dose of 15 mg/kg [22, 28]. The radiotracer also appears to be a substrate of mouse ABCG2, as evidenced by the significantly higher increase in brain uptake of [^18^F]AVT-011 following ABCB1 inhibition in *Abcg2*^−/−^ mice (8-fold) than in wild-type mice (3-fold). These results are consistent with functional redundancy between ABCB1A/B and ABCG2 at the blood-brain barrier in limiting brain uptake of dual ABCB1/ABCG2 substrates [29], and with previous reports on the brain uptake of other ABC transporter substrate radiotracers [30, 31]. While [^18^F]AVT-011’s lack of selectivity may be undesirable for imaging transporter function in certain pathophysiological conditions [11], its dual substrate property may be beneficial for imaging MDR mediated by ABC transporters in tumors because ABCB1 and ABCG2 are often co-expressed [1], and can independently and additively contribute to MDR [32].

While the higher brain uptake of [^18^F]AVT-011 in wild-type and *Abcg2*^−/−^ mice after tariquidar administration could have resulted from changes in perfusion, this possibility is unlikely for two reasons. First, administration of tariquidar was shown to have no effect on cerebral blood flow in non-human primates and humans, as measured by [^15^O]H_2_O PET [33, 34], suggesting that it will likely not affect cerebral blood flow in mice. Second, the lack of changes in brain uptake of [^18^F]AVT-011 in tariquidar-treated *Abcb1a/b*^−*/*−^ mice argues against the possibility that tariquidar exerted an effect on perfusion and that brain uptake of [^18^F]AVT-011 was perfusion-dependent. The increased brain uptake of [^18^F]AVT-011 in wild-type and *Abcg2*^−/−^ mice after tariquidar administration therefore is most likely a result of ABCB1 inhibition and not enhanced perfusion.

### Imaging ABCB1 transporter function in tumors with [^18^F]AVT-011

Using PET imaging, we demonstrated that [^18^F]AVT-011 can measure ABCB1 function in a mouse model of drug-resistant breast tumors. This previously described tumor model [24, 25], in which tumors from *Brca1*^*−/−*^*;p53*^*−/−*^ mice are transplanted into wild-type mice, recapitulates clinically observed MDR. That is, *Brca1*^*−/−*^*;p53*^*−/−*^ tumors show initial sensitivity to chemotherapy, but eventually acquire resistance to docetaxel, doxorubicin, topotecan, and/or olaparib with concomitant increases in *Abcb1a/b* expression [24, 35] to the levels found in clinical tumors [1]. In this study, [^18^F]AVT-011 could discriminate between tumors expressing basal and high levels of ABCB1, as evidenced by the 32% reduced uptake in tumors with high levels of ABCB1 and by the 40% increase in tumor uptake following tariquidar administration. Since the percentage increase in radiotracer uptake in muscle was not significantly different between mice that received tariquidar mid-scan and those that did not, the enhanced uptake after tariquidar administration in tumors was most likely a result of ABCB1 inhibition and not enhanced perfusion. Tumor uptake of [^18^F]FDG was also not significantly different between basal and high groups, making it unlikely that other factors such as tumor vascularization could account for the differences in tumor uptake of [^18^F]AVT-011. Lastly, differences in radiotracer metabolism could not explain these differences, as the percentage of unchanged [^18^F]AVT-011 was not different between mice harboring tumors with basal levels of ABCB1 and those with high levels. Together, these results indicate that [^18^F]AVT-011 images ABCB1-mediated MDR in tumors, although it may also recognize other transporter-mediated MDR (e.g., by ABCG2) in tumors.

While the radiotracer discriminated between tumors expressing basal and high levels of ABCB1, it did not show a significant difference between basal and intermediate levels of ABCB1. What would be the minimum threshold of transporter expression needed for a radiotracer to be useful in detecting transporter-mediated MDR in vivo? The answer to this question is important for cancer treatment because profiling of mRNA expression has been recommended as a screening tool for MDR in the clinic [1]. In our study, relative to values in the basal group, *Abcb1a/b* mRNA expression was ~ 3-fold higher and ABCB1 protein levels were ~ 4-fold higher in the intermediate tumor group, while *Abcb1a/b* mRNA expression was ~ 521-fold higher and ABCB1 protein levels were ~ 13-fold higher in the high ABCB1–expressing group. By PET, we measured a ~ 15% difference in ABCB1 function between the basal and intermediate-ABCB1 groups, and a ~ 32% difference between the basal and high ABCB1–expressing groups. This result is similar to the 50% reduced uptake of [^11^C]verapamil in drug-resistant tumors [18] and to the 35% reduced uptake of [^99m^Tc]sestamibi in drug-resistant tumors expressing 8-fold higher ABCB1 protein levels [36]. Because function does not appear to correlate linearly with expression, one caveat for using imaging is that the threshold of sensitivity for detecting ABCB1 function in tumors is currently unknown. Nevertheless, despite the lower sensitivity of PET in distinguishing basal versus high-ABCB1 groups compared with mRNA profiling, PET imaging could be clinically advantageous for detecting MDR because it is non-invasive and can be used in whole body, longitudinal studies.

### Comparison of [^18^F]AVT-011 with other radiotracers for imaging ABC transporters in tumors

How does [^18^F]AVT-011 compare with the other substrate radiotracers, including [^99m^Tc]sestamibi, [^11^C]verapamil, and [^18^F]fluoropaclitaxel, used to image MDR mediated by ABC transporters in tumors? In terms of substrate selectivity, our results show that [^18^F]AVT-011 is likely a substrate of both ABCB1 and ABCG2, while [^99m^Tc]sestamibi is transported by both ABCB1 and multidrug resistance–associated protein 1 (ABCC1) [37], and (*R*)-[^11^C]verapamil is selective for ABCB1 over ABCG2 and ABCC1 [38]. [^18^F]AVT-011 and [^99m^Tc]sestamibi therefore offer the possibility to probe different biological mechanisms of MDR, although ABCB1 and ABCG2 tend to be co-expressed in tumors with MDR [1].

[^11^C]Tariquidar is another radiotracer that was developed to image ABCB1 density [21]. The radiotracer displayed moderately higher uptake in a mouse xenograft tumor model overexpressing ABCB1 than in ABCB1-negative tumors [20], presumably due to ABCB1 binding. However, conflicting evidence exists whether this radiotracer is transported by ABCB1 [22, 23], which may confound PET measurements of ABCB1 density and/or function. In contrast, [^18^F]AVT-011 showed lower uptake in mouse orthotopic tumors expressing clinically relevant levels of ABCB1 than in those with basal levels of ABCB1, and showed increased tumor uptake after ABCB1 inhibition, consistent with the behavior of an ABCB1 substrate.

In terms of pharmacokinetics, [^18^F]AVT-011 has stable uptake in tumors, reaching a plateau within 15 min after injection. This pattern of uptake has also been observed for other ABCB1 radiotracers [18, 36], and may be a result of mitochondrial [39] or lysosomal trapping [40]. While the uptake at later time points may be non-specific, the initial uptake is likely to reflect the activity of ABCB1 (and likely ABCG2) and therefore would only require short PET scans for quantitation [18]. [^18^F]AVT-011 is metabolically stable, as evidenced by < 10% metabolism in mouse liver. In this sense, the radiotracer is advantageous because [^11^C]verapamil and [^18^F]fluoropaclitaxel are metabolized > 50% within 30–40 min [17, 18]. However, because its primary mode of excretion is through the hepatobiliary system, [^18^F]AVT-011 would not be useful in imaging tumors located in organs such as the liver and gastrointestinal tract (whose cancers have high *ABCB1* and *ABCG2* expressions) because the high baseline values of uptake in these organs would make it difficult to distinguish uptake in tumor versus normal tissue [1].

### Potential clinical utility of [^18^F]AVT-011

Given its properties, [^18^F]AVT-011 would likely be useful for screening an important mechanism of drug resistance (i.e., ABC transporter-mediated) in select cancers, such as breast tumors or lymph node metastasis. Detecting ABC transporter function in patients using a generic radiotracer such as [^18^F]AVT-011 may also offer clinicians the ability to select or avoid chemotherapeutics that are substrates for ABCB1/ABCG2. Furthermore, because of the ^18^F label, the radiotracer could potentially be distributed from a central production site to different hospitals without a radiochemistry facility, thereby allowing broader clinical use.

## Conclusion

We evaluated the pharmacological properties of a new PET radiotracer [^18^F]AVT-011. Using PET imaging in mice, we found that it is a substrate for mouse ABCB1 and ABCG2, is minimally metabolized, and undergoes primarily hepatobiliary excretion. [^18^F]AVT-011 PET measures ABCB1 function in tumors expressing clinically relevant levels of ABCB1 and could potentially be used to detect MDR in select human cancers.

## Electronic supplementary material

ESM 1 (DOCX 354 kb)
