# Supplementary material for: In vivo characterization of [18F]AVT-011 as a radiotracer for PET imaging of multidrug resistance
Source: Eur J Nucl Med Mol Imaging. 2019 Nov 15;47(8):2026–35. doi: 10.1007/s00259-019-04589-w (PMC7299908; doi:10.1007/s00259-019-04589-w)
Supplement: Supplementary file 1 — (DOCX 354 kb) [file 259_2019_4589_MOESM1_ESM.docx]

**SUPPLEMENTAL INFORMATION**

***In Vivo* Characterization [^18^F]AVT-011 as a Radiotracer for PET Imaging of Multidrug Resistance**

Pavitra Kannan, András Füredi, Sabina Dizdarevic, Thomas Wanek, Severin Mairinger, Jeffrey Collins, Theresa Falls, R. Michael van Dam, Divya Maheshwari, Jason T. Lee, Gergely Szakács, Oliver Langer

**supplementaL methods**

**Chemicals**

All chemicals were purchased from Sigma-Aldrich (Schnelldorf, Germany and St. Louis, MO, USA) and used without further purification. AVT-011 radiolabeling precursor (6-*O*-desmethly-tariquidar) was provided by Avaant Pharmaceuticals Inc and stored at -20°C. Aqueous [^18^F]fluoride was produced using a PETtrace cyclotron (GE Healthcare, Uppsala, Sweden) *via* the ^18^O(p,n)^18^F nuclear reaction by irradiation of a 2.6 mL water target containing 98% enriched ^18^O-water (Rotem Industries Ltd, Israel) with a 16.5 MeV proton beam. Tariquidar dimesylate (Haoyuan Chemexpress Co., Limited) was prepared fresh in a vehicle of DMSO, propylene glycol and 5% dextrose saline at a ratio of 2:2:1, or in 2.5% aqueous dextrose solution and administered intravenously (i.v.) to animals at a dose of 15 mg/kg.

**Radiosynthesis of [^18^F]AVT-011**

For brain uptake studies, radiosynthesis of [^18^F]AVT-011 was performed using the ELIXYS FLEX/CHEM radiosynthesizer (SOFIE, Inc., Culver City, CA, USA). After delivery of the irradiated ^18^O-water to the synthesis module, [^18^F]fluoride was trapped on a pre-conditoned anion exchange cartridge (QMA, ABX Advanced Biochemical Compounds (Radeberg, Germany). [^18^F]fluoride was eluted into the synthesis reactor by rinsing the cartridge with 1 mL of a 1:1 (v/v) mixture of 0.075M tetra-butyl ammonium bicarbonate (TBAHCO_3_) solution (dissolved in ethanol) and acetonitrile. The [^18^F]fluoride solution was evaporated under vacuum and a stream of nitrogen for 3 min at 110°C. Acetonitrile (1.2 mL) was then added to the reactor through the same elution line to rinse any residual activity into the reactor, and the solvent was evaporated azeotropically using the same conditions for 2 min. The azeotropic drying step was performed twice to ensure all water was removed. Ethylene di(p-toluenesulfonate) (7.0 mg, 18.9 µmol) dissolved in acetonitrile (1.0 mL) was added to the dried K-[^18^F]TBAF complex. The reaction mixture was heated at 75°C for 5 min and then cooled to 40°C. The precursor (2.0 mg, 3.2 µmol) and the base tetrabutylammonium hydroxide (14 µL of 40% TBAOH solution in water) were dissolved in dimethylformamide (0.5 mL), added to the reaction mixture, and heated at 100°C for 10 min. After cooling to 40°C, the mixture was passed over a silica Sep-Pak pre-activated with acetonitrile (10 mL) into a second reactor, where the solvent was evaporated under vacuum and a stream of nitrogen for 2-3 min at 100°C. The silica Sep-Pak was further rinsed 2 times with acetonitrile (3.0 mL each rinse); evaporation to dryness was performed after each transfer. Subsequently a mixture of water and acetonitrile (2 mL; 60:40 (v/v)) was added and the crude reaction mixture was injected into the semi-preparative high-performance liquid chromatography (HPLC) system equipped with a WellChrom K-501 HPLC pump (Knauer; Berlin, Germany), reversed-phase Luna column (5 µm, 10 x 250 mm, Phenomenex), ultraviolet (UV) detector (WellChrom Spectro-Photometer K-2501, Knauer), and gamma-radiation detector and counter (B-FC-3300 and B-FC-1000; Bioscan Inc.; Washington, DC, USA). Injection was performed using the ELIXYS HPLC injection valve connected to a 5 mL loop. The product was eluted at a flow rate of 5 mL/min with 60:40 (v/v) acetonitrile/water plus 0.1% TEA. The HPLC eluate was monitored in series for UV absorption at a wavelength of 254 nm and radioactivity. [^18^F]AVT-011, which eluted with a retention time of 13-15 min in a typical volume of 5 mL, was diluted with water (30 mL) and trapped on a C18 Sep-Pak Plus cartridge pre-activated with ethanol (5 mL) and water (10 mL). The cartridge was washed once with water (10 mL), followed by elution of [^18^F]AVT-011 with ethanol (2 mL). The ethanol was removed by evaporation at 85°C until completely dried and the final product was formulated for i.v. injection into rodents by adding 10% ethanol in phosphate buffered saline (v/v).

For tumor imaging, radiosynthesis of [^18^F]AVT-011 was performed in a custom-modified dual-reactor TRACERlab^TM^ FX_FDG_ synthesis module (GE Healthcare) comprising a small-volume V-shaped borosilicate glass reactor (3 mL). After delivery of the irradiated ^18^O-water to the synthesis module, [^18^F]fluoride was trapped on an anion exchange cartridge (QMA light, Waters Cooperation, Milford, MA) pre-activated with 0.5 M aqueous K_2_CO_3_ (5 mL) and water (15 mL). [^18^F]fluoride was eluted into the synthesis reactor by rinsing the cartridge with a mixture of kryptofix 2.2.2 (4,7,13,16,21,24-hexaoxa-1,10-diazabicyclo[8.8.8]hexacosane, 15 mg, 39.8 µmol) in acetonitrile (0.9 mL) and K_2_CO_3_ (3.5 mg, 25.3 μmol) in water (0.1 mL). After adding acetonitrile (0.8 mL) to the synthesis reactor, the solvent was evaporated azeotropically under vacuum, first for 5 min at 60°C and then for further 6 min at 120°C to remove the remaining water. Ethylene di(p-toluenesulfonate) (4.0 mg, 10.8 µmol) dissolved in acetonitrile (2.0 mL) was added to the dried K[^18^F]F-K_222_ complex. The reaction mixture was heated at 80°C for 5 min and then cooled to 40°C. Labeling precursor 6-*O*-desmethyl tariquidar (2.0 mg, 3.2 µmol) and the base tetrabutylammonium hydroxide (14 µL of TBAOH 30-hydrate, 234.0 mg, 292.5 µmol dissolved in 2.0 mL methanol) were dissolved in dimethylformamide (0.6 mL), added to the reaction mixture, and heated at 100°C for 11 min. After cooling to 40°C, the mixture was passed over a silica Sep-Pak pre-activated with acetonitrile (10 mL) into a second reactor, where the solvent was evaporated under vacuum for 5 min at 100°C. The silica Sep-Pak was washed 3 times with acetonitrile (2.0 mL); evaporation to dryness was performed after each transfer. Subsequently a mixture of water and acetonitrile (2 mL; 50/50 (v/v)) was added and the crude reaction mixture was injected into the built-in semipreparative HPLC system. A Merck Chromolith SemiPrep RP-18 column (10 x 100 mm, 10 µm) equipped with a Chromolith Semiprep RP-18 guard column (10 x 10 mm) was eluted at a flow rate of 5 mL/min for the first 5 min with a mixture of water and acetonitrile (80/20, v/v) followed by an increase of the acetonitrile percentage to 90%. The HPLC eluate was monitored in series for radioactivity and UV absorption at a wavelength of 254 nm. [^18^F]AVT-011, which eluted with a retention time of 17-18 min in a volume of 5 mL, was diluted with water (100 mL) and passed over a C18 Sep-Pak plus cartridge pre-activated with ethanol (5 mL) and water (10 mL). The cartridge was washed once with water (10 mL), followed by elution of [^18^F]AVT-011 with ethanol (3 mL). The ethanol was removed by evaporation on a rotary evaporator and the final product was formulated for i.v. injection into rodents by adding 10% ethanol in phosphate buffered saline (v/v).

**Quality Control**

Radiochemical purity and molar activity of [^18^F]AVT-011 were determined with analytical HPLC using an Agilent 1260 system (Agilent Technologies Österreich GmbH, Vienna, Austria) consisting of a quaternary pump, an auto-sampler and a column oven. UV absorption was detected with an Agilent 1260 diode array detector at a wavelength of 254 nm and 280 nm in series with a Raytest “Ramona” detector (raytest Isotopenmessgeräte GmbH, Straubenhardt, Germany) for radioactivity detection. An Agilent Zorbax Eclipse XDB-C18 column (4.6 x 150 mm, 5 µm, Agilent Technologies), was isocratically eluted with a 50/50 (v/v) mixture of monobasic sodium phosphate buffer (25 mM NaH_2_PO_4_, pH 7.0) and acetonitrile at a flow rate of 2 mL/min. The identity of [^18^F]AVT-011 was verified by HPLC co-injection with an authentical standard of unlabeled AVT-011. Osmolality (mosmol/kg) of formulated [^18^F]AVT-011 solution was measured using a Wescor Vapro 5520 Pressure Osmometer (Wescor Inc., Logan, USA).

**Quantitative analysis of PET data**

For brain uptake studies, G8 images were reconstructed using maximum-likelihood expectation maximization, Inveon images were reconstructed using 3D ordered subset expectation maximization with 2 iterations followed by maximum a posteriori with 18 iterations, and Focus 220 images were reconstructed using 3D filtered back-projection. All PET procedures were followed with CT scans (G8 PET/CT or CrumpCAT microCT (*1*)) for anatomical co-registration. PET images were decay-corrected to time of injection, normalized to units of percent injected dose per gram (%ID/g), co-registered to CT, and analyzed using AMIDE version 1.0.5 (*2*). To determine [^18^F]AVT-011 time-activity curves (TACs) for each mouse, a region of interest (ROI) was drawn for the whole brain of each CT image and used to quantify the corresponding PET image. ROIs were used to generate volumes of interest (VOIs), which were then transferred to the PET images of the individual time frames. TACs, expressed as % ID/g were calculated for each VOI.

For tumor uptake analysis, dynamic scans were binned into 2 x 23 frames, with a duration of 45 min each. Images were reconstructed using Fourier rebinning of the 3D sinograms followed by two-dimensional filtered back projection with a ramp filter, resulting in an image voxel size of 0.4 x 0.4 x 0.796 mm^3^. Data were normalized, and attenuation- and decay-corrected. Tumor ROIs were manually outlined over multiple planes in the static [^18^F]FDG scans using AMIDE software (*2*) and were mapped onto the dynamic PET scans to generate VOIs and TACs, as described above.

***Ex vivo* experiments**

**Metabolite and biodistribution analysis**

Radiometabolism and biodistribution of [^18^F]AVT-011 were measured *ex vivo* 30 min after injection of [^18^F]AVT-011. Wild-type, *Abcb1a/b*^-/-^ and *Abcg2*^-/-^ mice were injected under 2% isoflurane anesthesia *via* the tail vein with 28.5 ± 5.3 MBq of [^18^F]AVT-011. Following an uptake period of 30 min, animals were killed. Organs were removed, weighed, and measured in a gamma-counter. For metabolite analysis in liver, tissue was homogenized in RIPA buffer (ThermoFisher Scientific) and incubated on ice for 30 min. Methanol (10:1 solvent:RIPA) was added before lysates were centrifuged (10000 x g, 10 min, 4 °C). Supernatants were filtered (PVDF syringe filter, 0.2 um, Fisherbrand), concentrated via partial solvent evaporation (nitrogen 4 psi, full vacuum) in an ELIXYS reaction vial at 50 °C until volume was < 200 µL, and injected onto radio-HPLC for analysis (Phenomenex C18 Luna column, 5 µm, 60:40 acetonitrile: water containing 0.1% tetraethylammonium, 1.5 mL/min).

For metabolite analysis in plasma, blood samples were centrifuged to obtain plasma, which was subsequently precipitated by the addition of acetonitrile (1 µL per 1 µL plasma). The homogenate was vortexed and then centrifuged (12000 x g, 5 min, 21°C). The supernatant or diluted tracer solution (3 µL) were spotted on thin-layer chromatography (TLC) plates (silica gel 60F, 20 × 20 cm; Merck, Darmstadt, Germany), which were developed in ethyl acetate/ ethanol (8/2, v/v). The TLC plates were placed on multisensitive phosphor screens (Perkin-Elmer Life Sciences, Waltham, MA) and scanned at 300 dpi resolution using a Cyclone^®^ storage phosphor system (Perkin-Elmer Life Sciences). TLC data were analyzed using OptiQuant 5.0 software (Perkin-Elmer Life Sciences). For biodistribution studies, radioactivity counts in whole organs was measured on a gamma counter and decay-corrected.

**Determination of mRNA expression and protein levels in tumors**

Frozen tumor samples used for transplantation were pulverized under liquid nitrogen and homogenized in TRIzol™ Reagent (Life Technologies). Total RNA was isolated from tissue samples using Direct-zol® MiniPrep kit (Zymo Research) according to the manufacturer's guidelines. In-column DNAse I treatment was applied to prevent DNA contamination. cDNA samples were prepared from 300 ng total RNA using the Promega Reverse Transcription System Kit. The Pre-Developed TaqMan® assay Actin β (Actβ) (Life Technologies) was used as endogenous control in real-time PCR (RT-PCR) experiments; for quantifying *Abcb1a* and *Abcb1b* mRNA levels the respective TaqMan® primers were used. RT PCR analyses were carried out using the StepOne™ Real-Time PCR System (Life Technologies); mRNA fold differences relative to basal P-gp group were determined using the 2^−ΔΔCt^ method.

For Western blotting, samples were treated as previously described(*3*). Frozen tissue samples were thawed, sonicated (4×10 sec) and centrifuged at 13,000 rpm at 4°C for 15 min. Following quantification (Qubit Protein Assay Kit), protein lysates were re-suspended and incubated in Laemmli buffer for 5-10 min as described(*4*). Proteins were separated by SDS-PAGE and transferred to polyvinylidene difluoride membranes. ABCB1 expression was detected using a rabbit antibody (EPR10364-57, Rb, abcam) and quantified using densitometry analysis (ImageJ, NIH, Bethesda, MD, USA).

**supplementaL figures and tables**


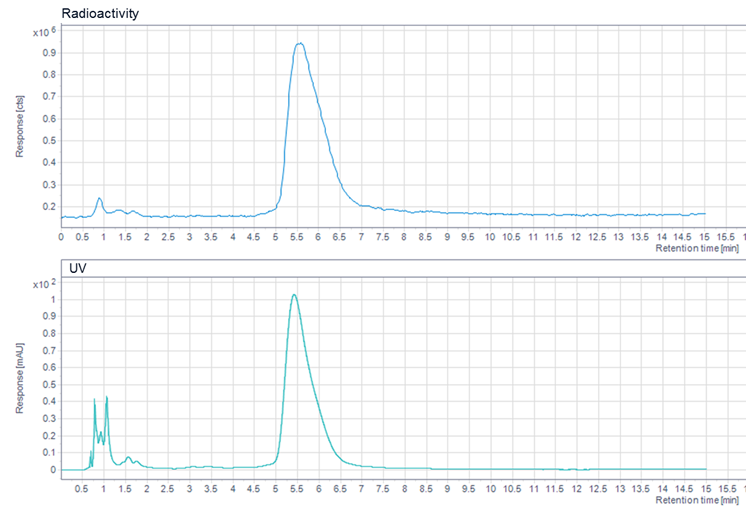


**[^18^F]AVT-011**

**AVT-011**

**Supplemental Fig.** **1** Representative analytical HPLC chromatogram of [^18^F]AVT-011 co-injected with unlabeled AVT-011. Upper trace represents radioactivity detection and lower trace represents UV absorption. See Supplemental Methods for employed analytical HPLC conditions (AIT method).


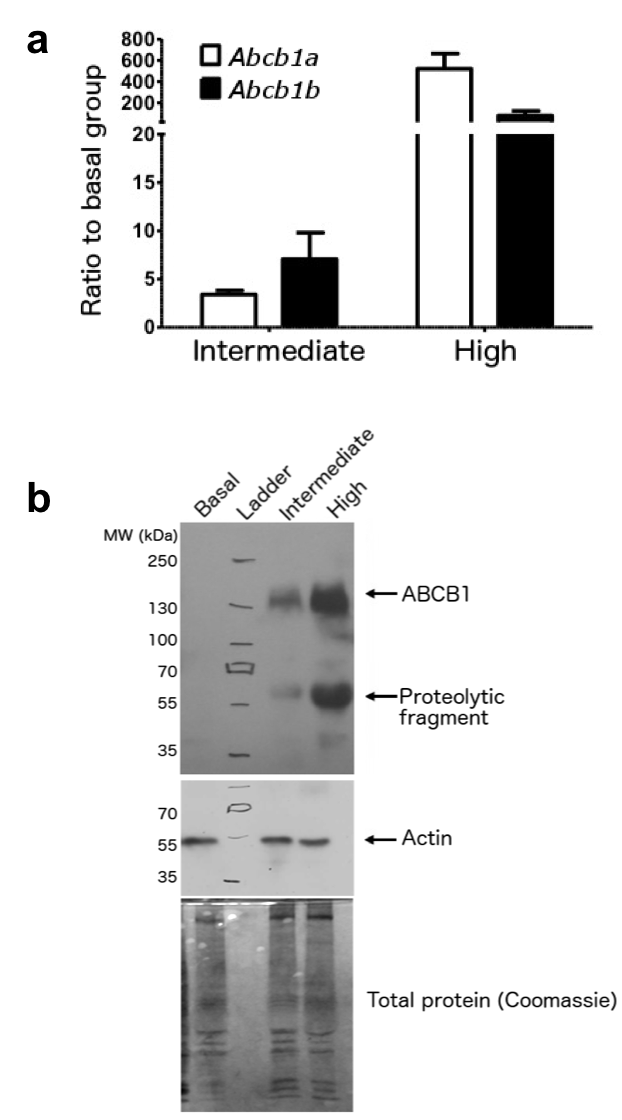


**Supplemental Fig. 2** Tumor pieces used for orthotopic transplantation have expected differences in (a) mRNA expression of *Abcb1a* and *Abcb1b* and (b) levels of ABCB1, Actin, and total protein. Bars represent mean ± SEM from n = 5 independent samples (basal and intermediate) and n = 3 (high). For Western blot, 100 μg of protein was loaded for each sample.


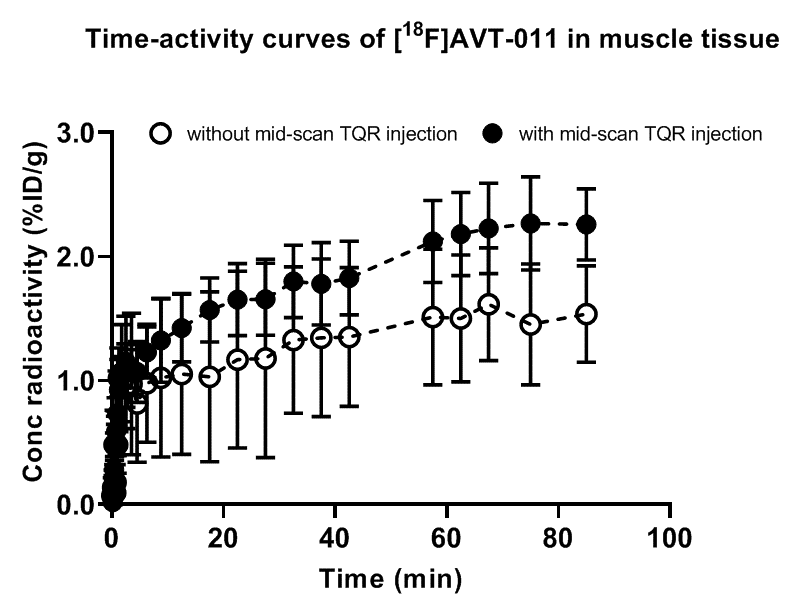


**Supplemental Fig. 3** Uptake of [^18^F]AVT-011 in muscle tissue is not enhanced by mid-scan tariquidar injection, as assessed by PET imaging. (a) Radiotracer uptake in muscle tissue (% injected dose, (ID)/g) after intravenous injection of [^18^F]AVT-011 in mice that received no tariquidar (TQR) mid-scan (open circles) or received 15 mg/kg tariquidar (black circles) mid-scan. Symbols represent mean ± SD from n = 3 mice/group.

**Supplemental Table 1.**

Uptake of Radioactivity (%ID/g) measured *Ex Vivo* in Various Organs of three Strains of Mice at 30 min after Injection of [^18^F]AVT-011

|  |  |  |  |  |  |  |  |  |  |  |  |  |  |
| --- | --- | --- | --- | --- | --- | --- | --- | --- | --- | --- | --- | --- | --- |
|  |  | Organ uptake (%ID/g) | | | | | | | | | | | |
| Organ |  | Wild-type | | |  | *Abcb1a/b*^-/-^ | | |  | *Abcg2*^-/-^ | | |  |
| Blood (whole) |  | 1.3 | ± | 0.4 |  | 1.3 | ± | 0.4 |  | 1.2 | ± | 0.2 |  |
| Bone |  | 1.5 | ± | 0.3 |  | 2.4 | ± | 1.0 |  | 1.5 | ± | 0.1 |  |
| Brain |  | 0.4 | ± | 0.5 |  | 0.8 | ± | 0.6 |  | 0.3 | ± | 0.0 |  |
| Heart |  | 3.6 | ± | 1.6 |  | 5.1 | ± | 1.0 |  | 7.2 | ± | 1.4 |  |
| Kidney |  | 14.1 | ± | 5.3 |  | 13.7 | ± | 5.1 |  | 23.2 | ± | 8.2 |  |
| Liver |  | 20.4 | ± | 6.6 |  | 22.9 | ± | 4.9 |  | 30.0 | ± | 8.0 |  |
| Lung |  | 15.7 | ± | 7.7 |  | 26.2 | ± | 11.6* |  | 28.8 | ± | 5.9** |  |
| Muscle |  | 1.0 | ± | 0.3 |  | 1.6 | ± | 0.7 |  | 1.2 | ± | 0.4 |  |
| Pancreas |  | 4.4 | ± | 0.5 |  | 5.8 | ± | 1.6 |  | 6.6 | ± | 2.7 |  |
| Small Intestine |  | 22.7 | ± | 14.6 |  | 19.3 | ± | 2.3 |  | 17.6 | ± | 3.2 |  |
| Spleen |  | 13.4 | ± | 5.7 |  | 6.4 | ± | 4.0 |  | 12.5 | ± | 2.2 |  |
|  |  |  |  |  |  |  |  |  |  |  |  |  |  |

Data represent mean ± SD from n = 3 mice for wild-type, n = 4 mice for *Abcb1a/b*^-/-^_,_ and n = 2 mice for *Abcg2*^-/-^_._ **P* < 0.05 and ***P* < 0.01 using 2-way ANOVA followed by Dunnett’s multiple comparison test (against wild-type).

**SUPPLEMENTAL REFERENCES**

1. Taschereau R, Vu NT, Chatziioannou AF. Calibration and data standardization of a prototype bench-top preclinical CT. In: IEEE Nuclear Science Symposium and Medical Imaging Conference (NSS/MIC). IEEE; 2014:1-2.

2. Loening AM, Gambhir SS. AMIDE: a free software tool for multimodality medical image analysis. *Mol Imaging*. 2003;2:131-7.

3. Pang H. Protein Extraction from Mice Xenograft Tumor. *BIO-PROTOCOL*. 2012;2.

4. Bársony O, Szalóki G, Türk D, et al. A single active catalytic site is sufficient to promote transport in P-glycoprotein. *Sci Rep*. 2016;6:24810.
